# End-to-End Automatic Classification of Retinal Vessel Based on Generative Adversarial Networks with Improved U-Net

**DOI:** 10.3390/diagnostics13061148

**Published:** 2023-03-17

**Authors:** Jieni Zhang, Kun Yang, Zhufu Shen, Shengbo Sang, Zhongyun Yuan, Runfang Hao, Qi Zhang, Meiling Cai

**Affiliations:** 1 Shanxi Key Laboratory of Micro Nano Sensor & Artificial Intelligence Perception, College of Information and Computer, Taiyuan University of Technology, Taiyuan 030024, China; ineijgnahz@163.com (J.Z.); sunboa-sang@tyut.edu.cn (S.S.); yuanzhongyun@tyut.edu.cn (Z.Y.); txlhrf@163.com (R.H.); zhangqi0564@link.tyut.edu.cn (Q.Z.); 2 Shanxi Institute of 6D Artificial Intelligence Biomedical Science, Taiyuan 030031, China; 3 Key Laboratory of Advanced Transducers and Intelligent Control System, Ministry of Education, Taiyuan University of Technology, Taiyuan 030024, China; 4 Department of Geriatrics, 920th Hospital of Joint Logistics Support Force, Teaching Hospital of Kunming Medical University, Kunming 650032, China; 5 Speed Electronics Company Limited, Room 305, Tower A, Luohutouzikonggu Building, Qingshuihe 1st Road, Luohu, Shenzhen 518000, China; 6 Image and Intelligence Laboratory, College of Information and Computer, Taiyuan University of Technology, Taiyuan 030024, China; caimeiling0570@link.tyut.edu.cn

**Keywords:** retinal vessel classification, fundus image, GAN, U-Net, image segmentation

## Abstract

The retinal vessels in the human body are the only ones that can be observed directly by non-invasive imaging techniques. Retinal vessel morphology and structure are the important objects of concern for physicians in the early diagnosis and treatment of related diseases. The classification of retinal vessels has important guiding significance in the basic stage of diagnostic treatment. This paper proposes a novel method based on generative adversarial networks with improved U-Net, which can achieve synchronous automatic segmentation and classification of blood vessels by an end-to-end network. The proposed method avoids the dependency of the segmentation results in the multiple classification tasks. Moreover, the proposed method builds on an accurate classification of arteries and veins while also classifying arteriovenous crossings. The validity of the proposed method is evaluated on the RITE dataset: the accuracy of image comprehensive classification reaches 96.87%. The sensitivity and specificity of arteriovenous classification reach 91.78% and 97.25%. The results verify the effectiveness of the proposed method and show the competitive classification performance.

## 1. Introduction

According to the “World Vision Report” issued by WHO, at least 2.2 billion people in the world are visually impaired or blind, and nearly half of the visual impairments could be avoided through early prevention [1]. By 2020, the number of glaucoma patients reached 796 million worldwide, and age-related macular degeneration patients numbered 200 million people [2,3]. The number of patients with diabetic retinopathy is expected to reach 200 million by 2035 [4]. However, the noted irreversible blinding diseases can be prevented and treated in advance by an ophthalmologist’s examination of the ocular fundus [5]. In this relevant examination, the pattern and structure of retinal vessels are important clinical characterizations. In addition, retinal blood vessels in the human body are the only blood vessels that can be directly observed with non-invasive imaging technology. The morphology is also affected by various factors of cardiovascular disease, hypertension, arteriosclerosis, and other systemic diseases [6,7,8]. The changes in retinal vessels, including vascular caliber, branch morphology, and arteriolar-to-venular diameter ratio (AVR), can be used as the diagnostic basis for vascular related diseases. For example, the method of observing the aspect ratio of retinal arterioles and the asymmetry of venous branches is an early monitoring method for Alzheimer’s disease [9]. The risk value of coronary heart disease and other diseases is also associated with the ratio of arteriovenous diameters [10]. In addition, arterial stenosis in the cerebrovascular network is significantly correlated with retinal arteriolar diameter [11].

Therefore, it is of great significance to classify the retinal vessels accurately for the prevention and observation of many diseases. However, early retinal vessel segmentation and classification mainly rely on manual labeling by professional doctors [12], which requires a lot of time and effort. Moreover, due to the reliance on subjective criteria, the results of segmentation and classification can be different. Automatic segmentation and classification of retinal vessels can greatly reduce the workload of doctors and can also avoid the impact of different doctors’ subjective factors on the classification results. With the development of computer vision technology, there are many methodologies for retinal vessel segmentation, including image filtering technology [13,14], machine learning algorithm for feature extraction [15], and neural network research [16]. However, related work that is dedicated to the classification of arteriovenous vessels is significantly less than that on vascular segmentation [17].

U-shaped convolution network (U-Net) [18] is widely used in the field of fundus blood vessel segmentation because of its excellent effect in the field of medical image segmentation. Specifically, the U-Net network is superior to convolutional neural network (CNN) and the fully convolutional networks (FCN) networks in the field of retinal vessel segmentation [19]. At the same time, generative adversarial network (GAN) has also been widely used in the field of fundus images and has been proven to be beneficial for various tasks [20]. In recent years, the GAN network has made good progress in the field of medical image segmentation. Some proposed GAN also obtained high accuracy for retinal vessel segmentation [19]. In addition, the atrous spatial pyramid pooling (ASPP) module enables the network to expand the receptive field and capture multi-scale contextual information without increasing the complexity of the algorithm parameters. It also reduces the loss of detailed information, which makes the vascular feature information better preserved and can enrich the microvascular information. In the task of blood vessel segmentation and classification, the number of pixels in the background of the fundus image is far greater than the number of blood vessels. In order to make the network pay more attention to the generation of blood vessel pixels, the attention module is also designed to be added to the network structure. At the same time, residual connections are introduced into the downsampling process of the U-Net of a generator network to alleviate the problem of gradient disappearance and increase the sensitivity of the generator network to weight changes, which makes the generator improve the vascular classification effect. In addition, low contrast is a major obstacle to the retinal image in optical imaging [21]. Before network classification, the preprocessing operations need to be conducted to enhance the contrast of the data.

To summarize, the contributions and novelty of the present study are highlighted as follows:

1. A model based on GAN and improved U-Net is proposed for the automatic end-to-end classification of fundus arteriovenous vessels. The introduction of ASPP and attention modules can also improve the classification capability of the model. The classification results of the proposed model are highly competitive.
2. A local contrast enhancement method was used to preprocess the input images. Through preprocessing, the problems of low overall brightness and poor contrast between blood vessels and background of the original fundus image data were solved. The effectiveness of this method was verified by ablation experiments.
3. The proposed method allows simultaneous classification of vessel crossings in fundus images in addition to the classification of arterioles, which is innovative in the study of fundus vascular classification.

The experimental materials used in this study are described in detail in Section 3. The design of the network model and the experimental process are described in Section 4. The results obtained are reported in Section 5. Section 6 summarizes this article.

## 2. Related Work

Although extensive research has been carried out in the field of retinal vessel segmentation, little attention has been paid to the field of automatic classification of retinal vessels [22]. Based on the existing research, the methods of retinal artery and vein classification can be divided into two categories: traditional machine learning based methods and depth learning based methods.

(A) Traditional Machine Learning Based Methods

Manual features. In the research of vessel classification based on traditional machine learning methods, it is usually necessary to manually extract features and then classify arteries and veins. It is often accompanied by some post-processing steps. Sathananthavathi et al. [23] extracted features manually according to the morphological structure of retinal vessels; the BAT evolutionary algorithm and the random forest classifier were used for main feature determination and classification, respectively; and, finally, the post-processing was used at the bifurcation of retinal vessels. Srinidhi et al. [24] and Xu et al. [25] also used manual features combined with random forest classifier to classify arteries and veins. Vázquez et al. [26] used the optic disc to divide the retinal vessels into many segments, and then the vessel segments were classified by color information, and the final classification result of whole blood vessel was determined by voting of the connected blood vessel segments.

Graph-based methods. Welikala et al. [27] avoided the use of hand-crafted features. The vascular network was first segmented from the retinal image. Bifurcations and crossover points were searched based on the retinal vascular skeleton, and vessel segments were segmented using the centerline. Finally, the vessel segments were fed into a convolutional neural network based on three convolutional and three fully connected layers to achieve the arteriovenous classification of retinal vessels. The classification rate with 47 features (the largest dimension tested) using OLPP in their own ORCADES dataset is only 90.56%, and the classification rate in the public dataset DRIVE is 86.7%. Zhao et al. [28] constructed the graph through image segmentation, skeletonization, and identification of significant nodes. They formalized the topology estimation and A/V classification into a pairwise clustering problem. The classification of blood vessels was effectively realized.

(B) Deep Learning Methods

Segmentation first and then classification. With the continuous development of deep learning, convolutional neural network has also been applied in the field of retinal vessel classification. Especially after U-Net [18] was proposed, it has performed well in the field of retinal vessel segmentation and classification, which leads to the realization of pixel-level segmentation and classification of fundus images. Li et al. [29] regarded arteriovenous classification as a three-classification task. First, the fundus image was preprocessed using the fuzzy removal technology, and then the image was classified by using the improved U-Net network. In order to improve the classification accuracy, the tracking algorithm was used as the post-processing method to further classify the blood vessels. Binh et al. [30] also regarded arteriovenous classification as a three-classification task, the improved U-Net model was used to classify retinal vessels, and the method of graph cutting was used for post-processing; the accuracy of their method is about 97.6%.

End-to-end classification. Morano et al. [31] decomposed the joint task into three segmentation problems: arteries segmentation, veins segmentation, and vessel segmentation. Their classification network consisted of the straightforward application of an FCNN with a custom loss. The accuracy of classifying retinal vessels reached 95.45%. Galdran et al. [32] used CNN as a task classification network. The previously segmented vascular tree did not need to be included in their classification method. Fully automatic classification of retinal blood vessels was achieved. They also proposed a classification of uncertain blood vessels. For the benefit of retrieval, the investigation of comparison is concisely summarized in Table 1.

## 3. Experimental Materials and Preprocessing

### 3.1. Dataset

**Figure 1:**
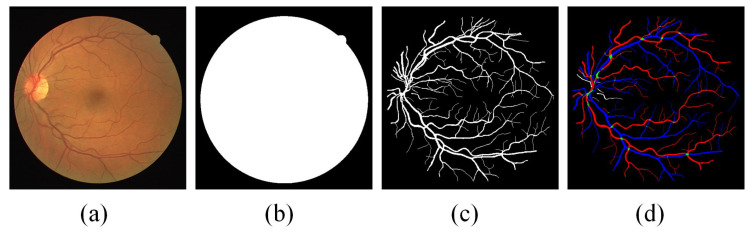
Examples of RITE dataset: (**a**) fundus images; (**b**) ROI mask; (**c**) ground truth of vessel segmentation; (**d**) ground truth of vessel classification.

The Retinal Images vessel Tree Extraction (RITE) dataset [33] is used in this work, which is derived from the DRIVE dataset [34]. It has been widely used as evaluation criteria in research fields such as retinal vessel segmentation, vessel extraction, and vessel classification. As shown in Figure 1, the RITE dataset is composed of four parts: fundus image, mask image used to extract the region of interest, the vessel trees manually segmented, and the A/V reference standard. The A/V reference standard is generated by marking each vascular pixel. In Figure 1d, red represents artery (A), blue represents vein (V), green represents crossing parts of artery and vein (O), and white represents uncertain vessel (U), with a resolution of 565 × 584 pixels.

### 3.2. Preprocessing

The collection process of image datasets inevitably has problems such as uneven lighting and noise. Similarly, the fundus image dataset is limited by imaging conditions such as low overall illumination and low contrast between blood vessels and background. These problems will have negative impacts on the further classification of fundus images. Therefore, this paper uses adaptive contrast enhancement (ACE) [35] to preprocess the original retinal fundus image.

The ACE algorithm divides the image into high frequency and low frequency. The low frequency part is obtained by smoothing, blurring, and other low-pass filtering methods. The high frequency part is directly obtained by subtracting the low frequency part from the original picture. In ACE, the high frequency part is amplified and added to the original low frequency part to obtain the enhanced image. The color constancy and brightness constancy of the enhanced image are improved, and the image contrast is changed. The details of ACE are as follows:

(1) $$y\left(i,j\right)=m_{x}\left(i,j\right)+G\left(i,j\right)\left[x\left(i,j\right)-m_{x}\left(i,j\right)\right],$$

where *x*(*i, j*) represents the pixel value corresponding to the image coordinate (*x, j*) before preprocessing; *$m_{x}$*(*i, j*) represents the low-frequency part, [*x*(*i, j*) −*$m_{x}$*(*i, j*)] represents the high-frequency part, and *G*(*i, j*) represents the high-frequency amplification coefficient (contrast gain).

The low-frequency part represents the local average value of the area with the window size of (2*n* + 1) × (2*n* + 1) centered on the image coordinate (*x, j*) pixel. The specific formula is as follows:

(2) $$m_{x}\left(x,j\right)=\frac{1}{{\left(2n+1\right)}^{2}}\sum_{k=i-n}^{i+n}\sum_{l=j-n}^{j+n}x\left(k,l\right)$$

According to the relevant research on ACE [36], the high-frequency amplification coefficient *G*(*i, j*) is defined as a variational constant, which is inversely proportional to the local mean square error, as shown in Equation (3).

(3) $$G\left(x,j\right)=\alpha \frac{\delta }{\sigma_{x}\left(x,j\right)},$$

where the value of δ is equal to the global mean square error of the image, which can reflect the dispersion of the image pixel value and the mean value. Constant α coefficient can linearly adjust the total amplification coefficient. The expression $\delta_{x}$ (*i, j*) represents the local mean square deviation, which can reflect the contrast change of the gray value of each pixel in the local area of an image. The expression of σ is shown in Equation (4).

(4) $$\sigma_{x}\left(x,j\right)=\sqrt{\frac{1}{{\left(2n+1\right)}^{2}}\sum_{k=i-n}^{i+n}\sum_{l=j-n}^{j+n}{\left[x\left(k,l\right)-m_{x}\left(x,j\right)\right]}^{2}}$$

**Figure 2:**
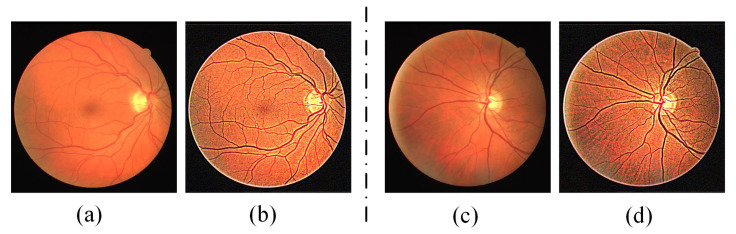
Two examples of fundus images before and after preprocessing: (**a**,**c**) original fundus image; (**b**,**d**) fundus image after preprocessing.

In addition, in order to avoid noise amplification or pixel value saturation caused by small local variance of the local part of image smoothing, the maximum value of *G*(*i, j*) is limited. The setting of the maximum of *G*(*i, j*) and the effects of all parameters is discussed in Section 5.5. The fundus images before and after preprocessing are shown in Figure 2. By comparing the original image, it can be seen that the contrast between the vascular pixels, especially the microvascular pixels and the background pixels, is significantly enhanced after preprocessing.

## 4. Methods

### 4.1. Method Architecture Overview

**Figure 3:**
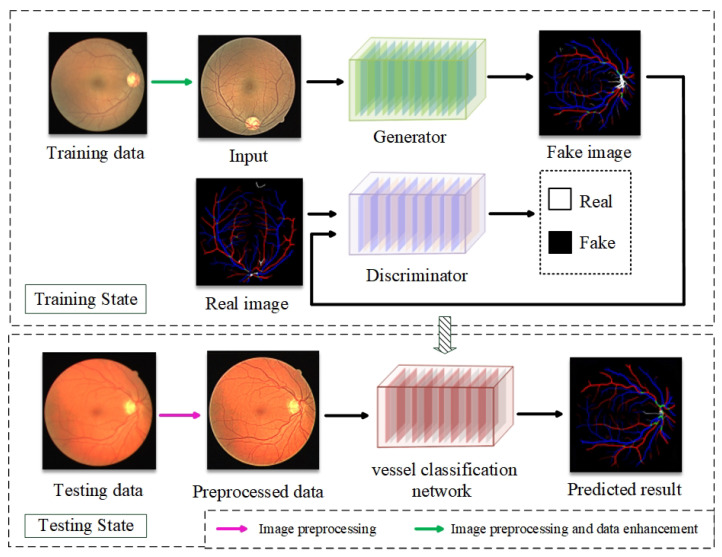
The overall flow chart of the proposed method.

The overall flow chart of the proposed method is shown in Figure 3. In the proposed method, ACE is used to preprocess the original retinal fundus image before performing network training and testing. The proposed model is designed using GAN. GAN is based on the idea of dynamic adversarial and can be divided into the generator and the discriminator parts. In this paper, the modified U-Net network is used as the generator and the convolutional network is used as the discriminator.

The generator is used to generate the vessel classification prediction result from the input training fundus image data, which is labeled as Fake Image. The corresponding Real Image is the ground truth of vessel classification from trained fundus image data. The discriminator is used to discriminate the image source, and the discriminated results are marked as Real and Fake. Being marked as Real means the image comes from the real vascular classification data Real Image, and being marked as Fake means the image come from the vascular classification data Fake Image, which is generated by the generator. By repeatedly training the generator and discriminator, the Fake Image will be as close to the Real Image as possible. When the discriminator cannot distinguish between real and false numbers, the required vessel classification network training is completed.

### 4.2. Network Structure

**Figure 4:**
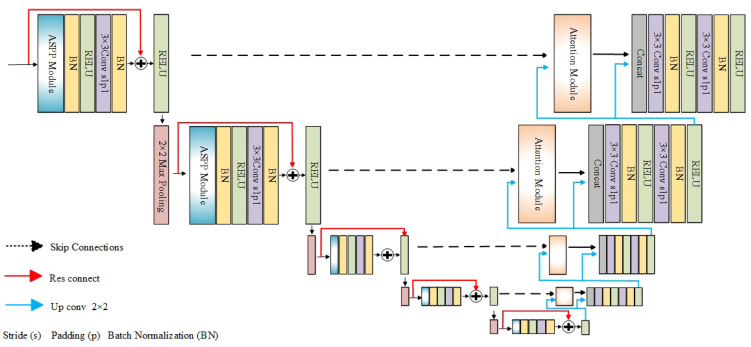
Improved U-Net network used as the generator (the ASPP Module is shown in Figure 6, and the Attention Module is shown in Figure 7).

The design details of GAN are described in this section. The U-Net [18] network is used as the main design of the generator. The structure of the designed generator network is shown in Figure 4. The atrous spatial pyramid pooling (ASPP) [37] module is added to the downsampling process of the U-Net network. The attention module is introduced at the skip link of the U-Net network. To alleviate the problem of gradient disappearance, residual connections are introduced into the downsampling process of the U-Net network. The residual connections helps to increase the sensitivity of the generator network to weight changes, which makes the generator fully learn the distribution of retinal vascular pixels and improve the vascular classification effect.

**Figure 5:**
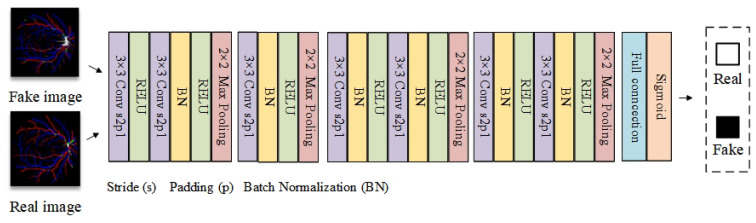
Discriminator network used.

The main role of the discriminator is to provide a descent gradient for the generator. Complex discriminator gradients will cause the gradient of the generator to disappear, which will not achieve effective adversarial training [38]. In this paper, ordinary convolutional networks are used as a discriminator, with the structure shown in Figure 5.

#### 4.2.1. ASPP Module

**Figure 6:**
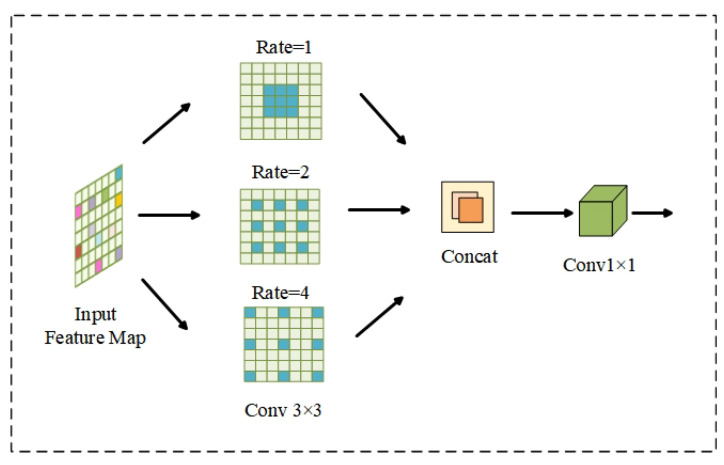
The structure of the ASPP module.

The original U-Net network uses downsampling to expand the receptive field while reducing the resolution, but the pooling layer of traditional convolution in U-Net will lose the details of retinal images, which leads to problems such as incomplete microvessel segmentation and susceptibility to breakage. In this paper, the ASPP module is added, which utilizes dilated/atrous convolution with multiple expansion rates to stack into a pyramidal structure instead of normal convolution. The structure of the ASPP module is shown in Figure 6. The structure enables the network to expand the receptive field and capture multi-scale contextual information without increasing the complexity of the algorithm parameters. It also reduces the loss of detail information, which makes the vascular feature information better preserved and can enrich the microvascular information.

#### 4.2.2. Attention Module

**Figure 7:**
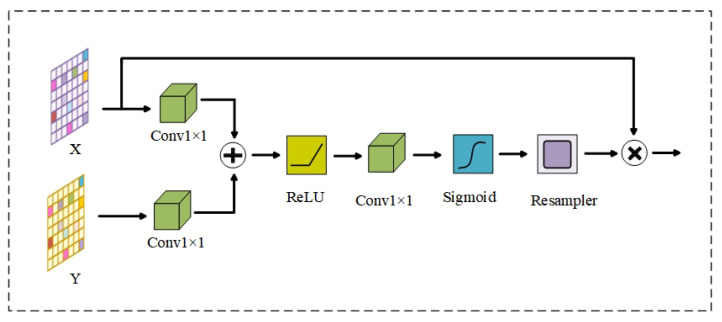
The structure of the attention module.

In the vascular classification task, the number of background pixels far exceeds the number of vascular pixels. The downsampled extracted feature maps are spliced directly with upsampling in the skip connection of traditional U-Net. Such a design produces a lot of redundant information and also leads to the deterioration of the extracted features. Inspired by Ashish Vaswani [39], the attention module is added to each skip to suppress excessive irrelevant information and make the model more concerned with the generation of vascular pixels. The details of the attention module are shown in Figure 7. In the figure, *X* is the feature map from downsampling and *Y* is the feature map from upsampling. After 1 × 1 convolution operation, *X* and *Y* are summed to highlight the features. After passing through ReLU and Sigmoid, the highlighted features are ranged between 0 to 1, which is the attention weight. It is assigned to the low-level feature after the attention weight is multiplied with *X*, and the attention allocation is achieved.

## 5. Experiments and Results

In this section, the experiments and results of the proposed method on the RITE dataset are detailed. Before going into the details of the implementation, the metrics to quantitatively evaluate the vascular classification results are described in Section 5.1. To ensure the validity of the proposed method, an ablation study is also described in Section 5.4. In addition, the effects of the parameters about the preprocessing are discussed in Section 5.5.

### 5.1. Evaluation Metrics

Considering the multiple cases of actual and predicted values in fundus images, a confusion matrix applicable to vascular classification studies is established, shown in Table 2. Because the proportion of arteriovenous crossing (A-V crossing) pixels and uncertain vessel pixels in fundus images is small, they are classified together as an uncertain vessel category.

According to the assessment methods widely used in the field of vascular classification [29,40,41,42,43], sensitivity (*Sens*), specificity (*Spec*), and accuracy (*Acc*) are used to assess the performance of the classification; the arteries are set as positive and the veins are set as negative. *Sens*, *Spec* and *Acc* are defined as follows:

(5) $$Sens=\frac{TA}{TA+FVa}$$

(6) $$Spec=\frac{TV}{TV+FAv}$$

(7) $$Acc=\frac{TA+TV}{TA+FVa+TV+FAv}$$

In order to quantitatively analyze the accuracy of uncertain vessels (including A-V crossing) classification, the index *Acc_U* is also introduced. To reflect a more comprehensive performance, the overall accuracy *Acc_ all* and the background segmentation accuracy *Acc_B* are added to evaluate the performance of the overall prediction of fundus images and the performance of the segmentation performance of non vascular pixels; *Acc_U*, *Acc_All,* and *Acc005FB* are defined as follows:

(8) $$Acc\underline{\;}U=\frac{TU}{TU+FVu+FAu}$$

(9) $$\begin{array}{c} Acc\underline{\;}All=\frac{TA+TV+TU+TB}{(TA+FAv+FAu+FAb+TV+FVa+FVu+FVb+TU+FUa+}\\ FUv+FUb+TB+FBa+FBv+FBu) \end{array}$$

(10) $$Acc\underline{\;}B=\frac{TB}{TB+FAb+FUv+FVb}$$

### 5.2. Implementation Details

Twenty color fundus images of RITE are selected randomly as the training set, and the remaining images are used as the test set. To increase the training data, a series of data enhancement operations such as horizontal flipping and rotating the images was performed. The network implementation is based on the Python language and the Pytorch framework, and the model is trained on a PC with a core i9-12900k CPU (3.8 GHz) and NVIDIA RTX3090-24GGPC (32 GB of RAM). During training, the batch_size was set to 1, the learning rate was 0.0008, and the optimizer was Adam. The experiment was trained for a total of 855 iterations and took about 7 h.

### 5.3. Classification Results

**Figure 8:**
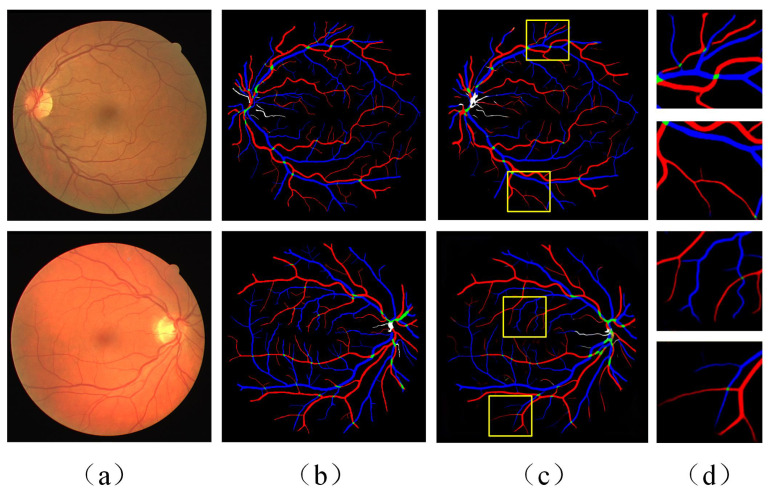
Example of vessel classification results of RITE fundus image dataset: (**a**) the original retinal fundus image; (**b**) ground truth of vessel classification; (**c**) vessel classification results of proposed method; (**d**) detail amplification of microvascular region.

The classification results of the proposed model on the RITE retinal images are presented in Figure 8. The validity of the proposed method is demonstrated by comparing the network prediction results of fundus images with the ground truth of vessel classification from the medical professional. In addition to the crude arteriovenous vessels, microvessels can also be more effectively and accurately classified. The details of the microvessel classification are magnified in Figure 8d. As described in Section 5.1, the results of the data quantified by the six evaluation metrics are shown in Table 3.

In Table 4, the experimental result is compared with the results of related studies based on the three important evaluation statistics of *Sens*, *Spec*, and *Acc*. As can be seen from Table 4, the proposed method outperforms most models in all evaluation metrics and achieves the highest scores on *Acc* and *Sens*. It shows the competitive performance of the proposed method compared to state-of-the-art methods. The evaluation metric *Sens* can be used to indicate arterial classification performance. The value of *Sens* of the proposed classification method increased to 91.78%, which proves the advantages of the proposed method in arterial classification performance. Among the methods, Morano et al. [31] designed an FCNN classification network with custom losses. Their method has a greater classification performance for veins. The value of *Spec* was increased to 98.67% in their method. However, the value of *Sens* only reached 78.07%. The balance between arterial classification and venous classification is the worst compared with other methods. The value of *Sens* also decreased by 13.71% compared with the proposed method.

**Figure 9:**
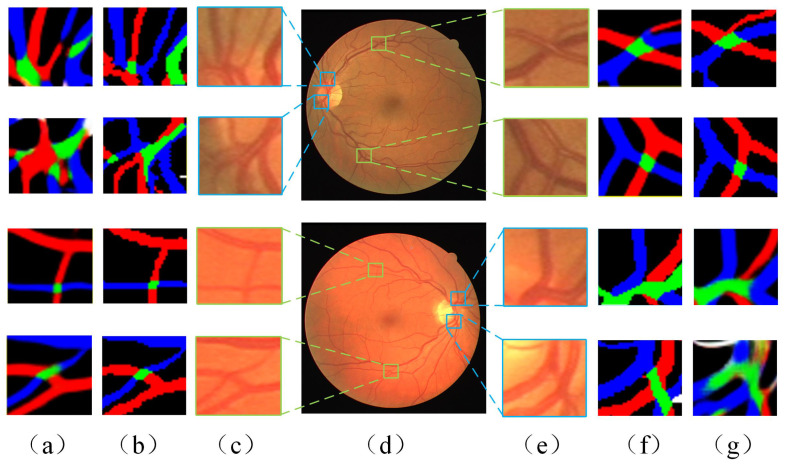
Details of A-V crossings classification results (the areas bounded by the blue rectangle is the examples near the optic disc, the areas bounded by the green rectangle is the examples far away from the optic disc). (**a**,**g**) A-V crossing classification results; (**b**,**f**) ground truth of A-V crossing; (**c**,**e**) fundal images of A-V crossing locally; (**d**) fundus image.

In the whole fundus image, the pixel of A-V crossings is a small proportion. As such, the corresponding classification training samples that can be obtained are few. Therefore the accurate classification of the A-V crossings is a greater challenge. In most current studies of fundus image classification [31,32,43,44,45], the identification of the A-V crossings region is not considered. However, A-V crossings are an area of concern for physicians. For example, the retinal crossover sign is one of the common alterations of retinal vessels in the fundus of hypertensive eyes [46]. The phenomenon of compression at the A-V crossings regions is the retinal arteriovenous crossing sign. When the A-V crossings are compressed, they are called retinal arteriovenous crossings signs. Therefore, A-V crossings also need to be considered in the classification studies of retinal vessels in the fundus images. The classification results of the A-V crossings are shown in Figure 9. The results show that A-V crossings far from the optic disc regions would enable a more accurate classification. However, due to the dense and complex distribution of blood vessels near the optic disc, the classification in these regions is not ideal.

### 5.4. Ablation Study

In order to verify the effectiveness of the proposed preprocessing and network improvement modules, six sets of ablation studies are conducted on the RITE dataset. The results of the ablation studies are shown in Table 5. The baseline model is the backbone network, where the original U-Net network is used as the generator network. Compared to baseline, adding residual connections *RS* improved Acc by 3.64% and Sens by 7.23%. After the addition of the ASPP module (*ASPPM*), *Acc* was improved by 3.10%, and *Sens*/*Spec* were improved, respectively, by 15.74%/3.02%. The addition of the attention module (*AttM*) increased *Acc*/*Sens*/*Spec* by 4.56%/14.66%/4.58%. Combining the residual connections, the ASPP module, results in an overall improvement for *Acc*/*Sens*/*Spec*/*Acc_U* by 4.91%/16.59%/5.54%/7.78%, which verified the effectiveness in the improved network. Moreover, with the preprocessing method (*Pre*), the results of *Acc*, *Sens,* and *Acc_U* reach the optimum. In summary, the proposed method is verified to have potential in the vascular classification task.

### 5.5. Discussion

**Figure 10:**
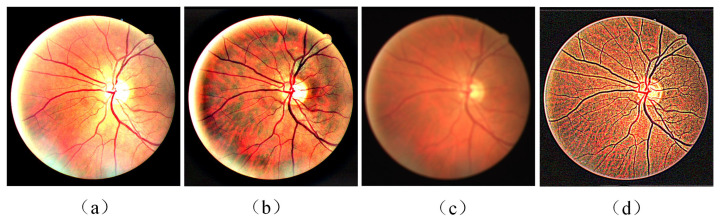
Examples of fundus images before and after preprocessing: (**a**) example of the overexposure phenomenon; (**b**) example of *n* equal to 50; (**c**) example of α equal to 0.01; (**d**) example of excessive noise.

In Equation (1), *G*(*x, j*) represents the high-frequency amplification coefficient (contrast gain). When it is a fixed constant, the high-frequency part is amplified in the same proportion. An overexposure phenomenon occurs in over-enhanced areas such as the edge of the fundus image and the center of the optic disc, as shown in Figure 10a. To avoid this phenomenon, the high-frequency amplification coefficient *G*(*x, j*) is defined as a variational constant in this work, which is inversely proportional to $\delta_{x}$ (*i, j*), as shown in Equation (3); $\delta_{x}$ (*i, j*) represents the local mean square deviation, and its value is directly related to *n*. Figure 10b shows an example of a result where *n* is equal to 50; the overexposure phenomenon at the edge of the fundus image and the center of the optic disc is significantly improved compared with Figure 10a. Due to the addition of $\delta_{x}$ (*i, j*), the high frequency amplification factor *G*(*x, j*) becomes a variable of spatial adaptation. In places where the image changes violently, the high-frequency amplification coefficient decreases accordingly, which can avoid the Ringing effect.

The function of α is to linearly adjust the value of *G*(*x, j*) to control the enhancement effect of the preprocessing. For example, the effect of α equal to 0.01 is shown in Figure 10c. In addition, since *G*(*x, j*) is inversely proportional to the local mean squared deviation, the local mean squared deviation may be small in areas where the image is smooth. If the local mean squared deviation is too small, and the *G*(*x, j*) will become large, which will lead to noise amplification or pixel saturation (i.e., pixel value exceeds 255). For example, this phenomenon can be observed easily, as shown in Figure 10d. When the noise is too large, the tiny vascular regions are greatly disturbed. Therefore, the maximum value of *G*(*x, j*) is limited to obtain better results. After comparing different preprocessing effects, this paper limits the maximum value of *G*(*x, j*) to 5.

**Figure 11:**
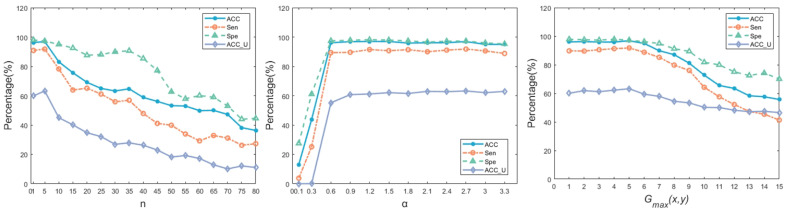
The parameter sensitivity analysis.

As a further detailed supplement, the parameter sensitivity analysis is shown in Figure 11. According to the analysis, the results show a slightly increasing trend when *n* is less than 6, and a significantly decreasing trend when *n* is greater than 6. When α is less than 0.9, it has a significant impact on the results, and when α is more than 0.9, it has a more subtle impact on the results. When the maximum setting is less than or equal to 5, for *Acc_U* has a slight improvement, and other results are slightly affected. When the maximum setting is greater than 5, all results basically show a downward trend.

### 5.6. Limitations

In fundus images, the pixel and background of blood vessels are not balanced, which is also a common problem in medical images. In particular, the classification of A-V crossings are involved in this work. Its content and background are seriously unbalanced, and vascular crossing needs further attention by the network. At the same time, the classification accuracy is greatly affected by the preprocessing effect, but the data preprocessing needs to rely on manual adjustment. In the experimental process, we found that when the number of epochs increases to a higher level, the classification performance for arterial vein and vascular crossing rapidly decline. In future work, better preprocessing methods and solutions to imbalance problems can improve the accuracy of classification of vascular crossing and achieve more stable performance.

## 6. Conclusions

In this paper, a method based on the combination of the GAN and U-Net networks is proposed for vessel classification of fundus images. This method preprocesses the input images by ACE to solve problems such as unclear data of original fundus images. In addition, the confusion matrix established in this study for vessel classification has reference value for comprehensive analysis and evaluation of prediction results. In the design of GAN, the original U-Net network is improved by introducing the residual connection, the null pyramid module, and the attention module. The proposed network structure not only achieves the end-to-end prediction for arterial and venous vessels, but also predicts the arterial–venous crosspoint region. Due to the complexity of the optic disc region and the small percentage of vascular cross-pixels, there is still much room for improvement in the accuracy of vascular cross-point recognition. Compared with the existing end-to-end methods, this study has improved the accuracy of vessel classification, which is important for retinal vessel classification to 96.86%. Experimental results show that the proposed method can effectively realize the automatic classification of arteriovenous and A-V crossings. This can be applied to the basic stage of screening and diagnosis of retinal vascular related diseases to improve the diagnostic efficiency of doctors, which has important significance for clinical practice. The proposed model design can also provide important reference for similar semantic segmentation tasks. In addition, the distribution of retinal vessels, arteriovenous intersection, and other characteristics have individual uniqueness, which have potential application value in biological recognition and other fields. In addition, some reliable future research can be continued based on the advantages and limitations of our research methods. For example, research directions to improve the accuracy of A-V crossings are needed. In addition, the fundus image enhancement method based on depth learning can be tried to solve the disadvantages of data optical imaging and improve the classification accuracy. Automated measurement of arteriovenous caliber ratios can be achieved based on arteriovenous classification, which has important implications for the diagnosis of diabetes. 

## Figures and Tables

**Table 1:** Summary of contemporary retinal vessel classification methods.

Method	Category	Pros and Cons	Examples
Traditional	Manual features	The extracted vascular features can be explained, but feature processing requires more professional knowledge of fundus vascular medicine and images	[23,24,25,26]
	Graph-based methods	The interpretable features can be extracted automatically, and the vascular segments are classified. However, accurate segmentation at the pixel level is not achieved	[27,28]
Deep learning	Segmentation first and then classification	The accuracy of classification is good, but the vascular classification task is divided into multiple subtasks, and the errors in the vascular segmentation subtask will be directly transmitted to the vascular classification subtask	[18,29,30]
	End-to-end classification	Automatic blood vessel segmentation and pixel-level classification are implemented synchronously, but the amount of related research is insufficient	[31,32]

**Table 2:** The confusion matrix for vascular classification of fundus images.

	Forecast Classification Results			
Actual Category	A (Artery)	V (Vein)	U (Uncertain Vessel)	B (Background)
**A (Artery)**	TA	FVa	FUa	FBa
**V (Vein)**	FAv	TV	FUv	FBv
**U (Uncertain vessel)**	FAu	FVu	TU	FBu
**B (Background)**	FAb	FVb	FUb	TB

**Table 3:** Vessel classification assessment results.

Formula	Index	Result	Comments
(1)	*Sens*	91.78%	The ability to classify arteries
(2)	*Spec*	97.25%	The ability to classify veins
(3)	*Acc*	96.86%	The ability to comprehensively classify the arteries and veins
(4)	*Acc_U*	63.23%	The ability to classify uncertain vessels (including A-V crossing)
(5)	*Acc_All*	96.87%	The ability to accurately classify the whole fundus image
(6)	*Acc_B*	98.88%	The ability to classify non-vascular regions

**Table 4:** Comparison of the assessment results for the related advanced arteriovenous classification.

Methods	*Acc*	*Sens*	*Spec*	Datasets	A-V Crossing	Years	End to End
Xu et al. [44]	92.30%	90.00%	90.00%	RITE	No	2017	No
Dashtbozorg et al. [25]	87.40%	73.00%	84.00%	DRIVE	No	2014	No
Kang et al. [41]	90.81%	88.63%	92.97%	RITE	No	2020	No
Huang et al. [45]	72.00%	70.90%	73.80%	DRIVE	No	2018	No
Galdran et al. [32]	89.00%	89.00%	90.00%	RITE	Yes	2019	Yes
Morano et al. [31]	96.05%	78.07%	**98.67%**	RITE	No	2021	Yes
This work	**96.86%**	**91.78%**	97.25%	RITE	Yes	2023	Yes

The best results are highlighted in bold.

**Table 5:** Ablation study results of baseline and improved network.

Methods			Vessel Classification					
Baseline	*RS*	*ASPPM*	*AttM*	*Pre*	*Acc*	*Sens*	*Spec*	*Acc_U*
Yes	No	No	No	No	91.13%	72.67%	92.33%	52.08%
Yes	Yes	No	No	No	94.77%	79.90%	96.61%	54.31%
Yes	No	Yes	No	No	94.23%	88.41%	95.35%	58.98%
Yes	No	No	Yes	No	95.69%	87.33%	96.91%	57.11%
Yes	Yes	Yes	Yes	No	96.04%	89.26%	97.87%	59.86%
Yes	Yes	Yes	Yes	Yes	96.86%	91.78%	97.25%	61.23%

## Data Availability

Not applicable.
